# A Three-Year Analysis of Toxic Benzene Levels and Associated Impact in Ploieşti City, Romania

**DOI:** 10.3390/toxics11090748

**Published:** 2023-09-02

**Authors:** Mia Sanda, Daniel Dunea, Stefania Iordache, Alin Pohoata, Ana-Maria Glod-Lendvai, Ion Onutu

**Affiliations:** 1 Faculty of Petroleum Processing and Petrochemistry, Petroleum-Gas University, Bulevardul București 39, 100680 Ploieşti, Romania; mia.sanda@upg-ploiesti.ro (M.S.); ionutu@upg-ploiesti.ro (I.O.); 2 Department of Environmental Engineering, Faculty of Environmental Engineering and Food Science, Valahia University of Targoviste, Aleea Sinaia no.13, 130004 Targoviste, Romania; ana.glod-lendvai@valahia.ro; 3 Department of Food Engineering, Faculty of Environmental Engineering and Food Science, Valahia University of Targoviste, Aleea Sinaia no.13, 130004 Targoviste, Romania; stefania.iordache@yahoo.com; 4 Faculty of Sciences and Arts, Valahia University of Targoviste, Aleea Sinaia no.13, 130004 Targoviste, Romania; alinpohoata@yahoo.com

**Keywords:** benzene, VOCs, petroleum refineries, lockdown, HYSPLIT, ExpoFIRST, ADD, LADD

## Abstract

This study examines the levels of benzene and the potential health impact during three years of continuous monitoring (2019–2021), including the COVID-lockdown period from 2020 in a city that is an important Romanian center for petroleum refining and associated product manufacturing. The dataset contains benzene, toluene, NOx, PM_10_ concentrations, and meteorological factors monitored by six automatic stations from the national network of which four are in the city and two outside. Special attention was given to the benzene dynamics to establish patterns related to the health impact and leukemia. An assessment of the exposure was performed using EPA’s ExpoFIRST v. 2.0 for computing the inhalation Average Daily Dose (ADD) and Lifetime Average Daily Dose (LADD). The health impact was estimated based on several indicators such as lifetime cancer risk (LCR), Hazard Quotient (HQ), Disability-Adjusted Life Years (DALY), and Environmental burden of disease (EBD). Overall, the annual average of all stations was almost similar between years i.e., 3.46 in 2019, 3.41 in 2020, and 3.63 µg/m^3^ in 2021, respectively. The average of all stations during the lockdown period was 2.67 µg/m^3^, which was lower than the multiannual average of the 2019–2021 period, i.e., 3.5 µg/m^3^. Significant correlations were present between benzene and other pollutants such as NOx (r = 0.57), PM_10_ fraction (r = 0.70), and toluene (r = 0.69), and benzene and temperature (r = −0.46), humidity (r = 0.28), and wind speed (r = −0.34). Regarding the ADD, in all scenarios, the most affected age categories are small children, despite a lower outdoor exposure time. From birth to <70 years, the ADD varied depending on the exposure scenario resulting in 3.27 × 10^−4^, 5.6 × 10^−4^, and 4.04 × 10^4^ mg/kg-day, and 3.95 × 10^−4^, 10.6 × 10^−4^, and 6.76 × 10^−4^ mg/kg-day for the LADD, respectively. The Integrated Lifetime Cancer Risk (ILTCR) values were 14.1 × 10^−5^ in winter, 9.04 × 10^−5^ in spring, 8.74 × 10^−5^ in summer, and 10.6 × 10^−4^ in autumn. The ILTCR annual averages were 1.08 × 10^−4^ (2019), 1.07 × 10^−4^ (2020), 1.04 × 10^−4^ (2021), and 1.06 × 10^−4^ for the entire period. The resulting ILTCR values point out very risky conditions, with the annual averages reaching the definite cancer risk category. The corresponding burden based on the DALY’s loss due to leukemia in Ploieşti was estimated at 0.291 (2 μg/m^3^ benzene), 0.509 (3.5 μg/m^3^ benzene), 0.582 (4 μg/m^3^ benzene), and 0.873 DALYs per 100,000 inhabitants (6 μg/m^3^ benzene), respectively. The current study provides useful insights for a better understanding of the exposure levels to benzene and associated health impact in Ploieşti despite the limitations determined by the data hiatus and incomplete or missing information regarding the health impact.

## 1. Introduction

Air pollution occurs when harmful substances are emitted into the air altering the air’s physical, chemical, and biological properties. Air pollution is one of the most serious environmental issues in terms of human health and the surrounding environment and is associated with significant economic burdens on the global economy because of premature death, diseases, lost earnings, and increased health care expenditures, all of which constrain productivity and economic growth [1].

Volatile organic compounds (VOCs) represent an important hazardous group of air pollutants [2,3]. In this group, BTEX is formed by benzene, toluene, ethylbenzene, and o-, p-, and m-xylene and is emitted to the atmosphere from both industrial and biogenic sources having carcinogenic and mutagenic effects [4]. Even at low concentrations, VOCs can easily enter the body through the air and, therefore, cause health risks during long-term exposure. From the BTEX group, benzene (C_6_H_6_) is more carcinogenic to humans, frequently favoring the occurrence of myeloid leukemia due to the myelotoxicity of benzene [5]. A lifetime exposure of urban populations to 1.7 μg/m^3^ benzene can cause 10 cases of leukemia per million inhabitants, which was estimated by the World Health Organization (WHO) [6]. In the European Union, the limit value for benzene for the protection of human health is the annual mean value that may not exceed 5 µg/m^3^ established in the air quality directive (2008/EC/50). The air quality standards for benzene in various regions and countries in the world are summarized by Sekar et al. [7].

The U.S. ATSDR stated that industrial processes are the main sources of benzene in the environment. Benzene levels in the air can be elevated by coal and oil burning emissions, benzene waste and storage operations, motor vehicle exhaust, and evaporation from gasoline service stations [8]. People may be exposed to higher levels of benzene in the air by living near hazardous waste sites, petroleum refining operations, petrochemical manufacturing sites, or gas stations [9]. It was found that slow degradation pollutants (i.e., benzene) accumulate in poorly ventilated areas, whereas faster degradation pollutants do not show accumulation [10], which is important for characterizing the urban morphology and optimal placement of the monitoring stations. According to the National Air Toxics Assessment (NATA) data, the leading source of benzene emissions to US ambient air is emissions from mobile on-road sources, which account for 49% of all emissions nationally and 57% of all emissions from urban areas. In outdoor conditions, the highest exposures to benzene could occur in heavy traffic, while filling vehicle gas tanks, and at or near gas stations (https://www.epa.gov/national-air-toxics-assessment (accessed on 8 August 2023)).

Previous research reported that petroleum refining can generate significant atmospheric emissions [11,12,13] including BTEX, particulate matter, and hydrogen sulfide (H_2_S). The sources of air emissions are represented by the pollutants associated with the technological processes and the generation of thermal energy, corresponding to the operating situation of a refinery. In addition to their toxic effect, from an oil refinery, they are created mainly by sulfur compounds (for example, hydrogen sulfide, mercaptans, sulfides, disulfides); nitrogen compounds (e.g., ammonia, amines); and hydrocarbons (e.g., various aromatic compounds).

In general, the installations, emission points, and equipment associated with the air polluting incidents in refineries are not included in the current analysis and monitoring a category of compounds present in the air that can be dangerous, even at low concentrations, for human health, namely: benzene, 1,3 butadiene, formaldehyde, and hydrogen sulfide [14].

It is possible, in some cases, to have differences of orders of magnitude between the actual and estimated emissions, depending on the particularities of the source configurations, control equipment, and operating practices. Thus, in all situations where an accurate emissions assessment of these pollutants is required, source-specific information should be obtained to confirm the existence of specific emitting operations and the types and effectiveness of control measures and determine the impact of operational practices. Source testing and/or material balance calculations should be considered as one of the optimal methods for determining these emissions.

Emissions of benzene, as well as emissions of other hazardous air pollutants (PAHs) from oil refineries, can be grouped into five main categories: (1) pipelines/vents from facilities, (2) storage tanks, (3) spills equipment, (4) transfer operations, and (5) wastewater collection and treatment. Due to the limited availability of data on the potential pollutant emission sources in the area and the variability of generating process configurations, control equipment, and operating procedures among facilities, this work considers the concentrations measured by the automatic stations from the Romanian National Monitoring Network [15]. Although benzene, 1,3 butadiene, formaldehyde, and hydrogen sulfide emissions are reported in the city of Ploieşti and its suburbs, an exact assessment of the emissions from a specific refinery/emission source cannot be made. This situation is because either the data is missing or those who received requests to report the level of these pollutants did not always provide complete answers.

In Romania, there are very few recent articles regarding the benzene outdoor levels in urban areas. When using the syntax “air pollution-benzene–Romania” for searching the Clarivate Web of Science database from 2015 to 2023, 10 articles were found, and only 3 present the situation of outdoor concentrations of various pollutants including benzene in several Romanian cities, i.e., Timisoara [16], Arad [17], and Ploieşti [18]. None of them analyzed the evolution of benzene after 2019, including the lockdown period, and no health impact assessment was found. Consequently, the current work presents a three-year analysis of the benzene level dynamics (2019–2021) and associated health impact based on hourly measurements trying to estimate the impact of petroleum refining and heavy traffic both in Ploieşti and adjacent neighboring areas that have functional refineries.

The main aim of this work is to identify, characterize, monitor, and localize the areas with high levels of benzene, looking for potential solutions to improve the air quality plan in Ploieşti City, on one hand, to reduce the emissions, and, on the other hand, to diminish the harmful effect on the residential population. The dataset contains concentrations monitored by six automatic stations from the national network between 1 January 2019 and 31 December 2021, including the COVID-19 lockdown in 2020. Moreover, useful insights regarding the synergy with other pollutants (PM_10_, toluene, and NOx) and meteorological parameters were included. 

The specific objectives of this work were to establish:

1. Differences between various locations of the city and its suburbs based on monitoring stations’ data acquisition;
2. Differences between the years based on aggregated time series (2019–2021);
3. Differences in concentrations during the COVID-19 lockdown in 2020;
4. Relationships with other pollutants and meteorological factors;
5. Existing benzene patterns and the potential impact on health in the Ploieşti area.

## 2. Materials and Methods

### 2.1. Study Area and Environmental Impact 

**Figure 1:**
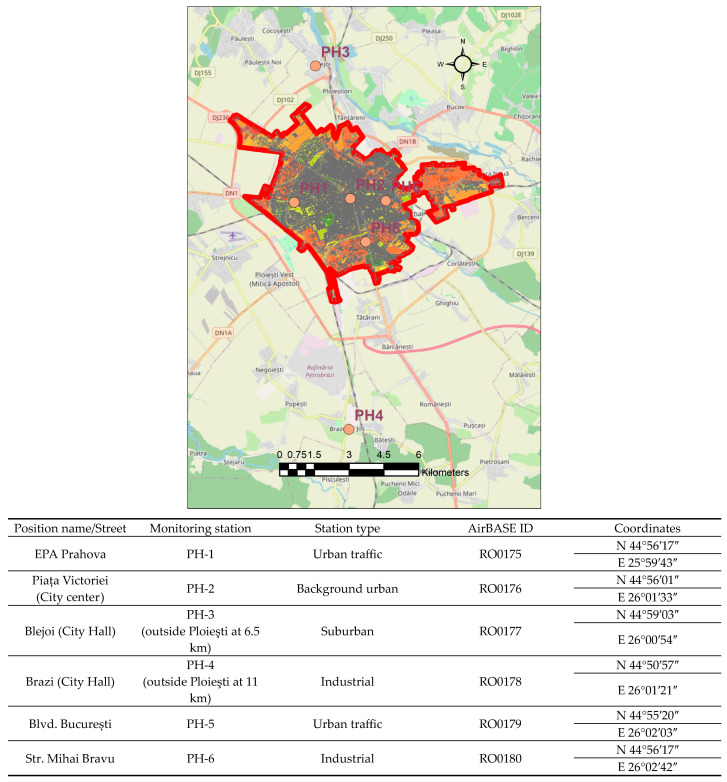
Map of Ploieşti City showing the positions of the official monitoring stations from the Romanian National Network of Air Quality Monitoring (RNMCA).

Ploieşti is one of the largest urban agglomerations in Romania, ranking in 10th place with 180,539 inhabitants according to the last census (2022), with a drop of 29,406 compared to the 2011 census (Figure 1). It is in the south-east of Romania (44°56′24″ N Lat.; 26°01′00″ E Long.; 150 m a.s.l.) and it is the largest city located near Bucharest (60 km), Romania’s capital. 

Ploieşti City is an important industrial center, which experienced rapid economic growth in the last decades. Its industrial activity is concentrated, especially in the oil production and refining industry. Ploieşti has four oil refineries in proximity, but only three are operating at different magnitudes. Even if the oil production in the region is declining steadily, there is still a significant processing industry that operates. The city remained anchored mainly in the oil processing industry and extractive industries related to this sector (construction equipment/building machinery, electrical equipment, metallic construction and metal products, chemicals and man-made fibers, manufacturing of rubber and plastic products, etc.). Unfortunately, the urban residential areas are located relatively close to the industrial facilities [18]. Due to its large population, Ploieşti City also has a heavy traffic density. Many commuters come to and leave the city every day, using private cars because of an undersized public transportation system [12]. These reasons lead to traffic congestion within the city center; more than 80,000 vehicles per day are transiting the inner city. Ploieşti is an important transport hub facilitating links to other regions of Romania (i.e., Moldavia and Transylvania). 

### 2.2. Climate and Meteorological Data

The climate of Ploieşti is influenced by its position in the Ploieştilor Plain. The air temperature has an annual average of 10.5 °C with a minimum temperature recorded on 25 January 1952 (−30 °C), while the maximum temperature was recorded on 19 July 2007 (43 °C). The multiannual precipitation quantity is around 600 mm (average values of 30–40 mm in January and 88 mm in June). 

The city is under the influence of predominant winds from NE (40%) and SE (23%), with an average speed of 3 m s^−1^. On average, there are 9 days/year with wind speeds over 10 m s^−1^ and only 2 days/year with wind speeds over 16 m s^−1^.

The lack of air currents, which is the state of atmospheric calm considering an average wind speed below 1.5 m s^−1^, generally amplifies the increase of the air pollutants’ concentrations including benzene.

Furthermore, temperature inversions occur in all the months of the cold season, when polar or arctic air enters the city, and a “dome” is formed under which the pollutants, stopped in their ascent, progressively concentrate, and disperse horizontally [19]. 

### 2.3. Monitoring Network and Data Analysis

The continuous monitoring of air quality is performed by 6 automatic stations (PH-1 to PH-6) distributed in the Ploieşti agglomeration and connected to the Romanian national Network for Monitoring Air Quality (RNMCA) (https://calitateaer.ro/public/home-page/?__locale=en (accessed on 8 August 2023)) complied with the European standards for air quality monitoring and assessments. Four of them (PH-1, PH-2, PH-5, and PH-6) are in the city, while the remaining (PH-3 and PH-4) are in adjacent areas near refineries, outside of Ploieşti city limits (Figure 1). 

The monitoring system allows the local authorities to protect the environment:

- To constantly evaluate, know and inform the public, other authorities, and interested institutions, about air quality status and trends;
- To take, in proper time, prompt measures to reduce or eliminate pollution episodes;
- To prevent accidental pollution;
- To warn and protect the population in case of emergency.

The values measured online by the sensors of the analyzers installed in the stations are transmitted via GPRS to the local centers. They are interconnected, forming a network that also includes the central servers where all the data arrives and from where it is brought to the attention of the public in real-time on the website: http://www.calitateaer.ro (accessed on 8 August 2023) and other means [20]. Data regarding the benzene concentrations were recorded hourly between January 2019 and December 2021, including the COVID-19 lockdown period in 2020.

Benzene (C_6_H_6_) is a very light aromatic compound, volatile and soluble in water. It is a carcinogenic substance, classified in the A1 toxicity class, producing harmful effects on the central nervous system. Approximately 90% of the amount of benzene in the ambient air comes from road traffic. The remaining 10% comes from fuel evaporation during its storage and distribution. Oil refineries contribute to this amount (https://www.epa.gov/sites/default/files/2016-09/documents/benzene.pdf accessed on 25 July 2023). 

All stations measure benzene following the reference method provided in the SR standard EN 14662-Ambient air quality. Standardized method for measurement of benzene concentration-parts 1, 2, and 3 (https://www.calitateaer.ro/public/assessment-page/pollutants-page/benzen-page/?__locale=en accessed on 25 July 2023) using the Orion VOC/BTX-2000 analyzer based on a detector with PID photoionization, EN 14662-1 (https://standards.iteh.ai/catalog/standards/cen/924ad2ff-37ac-478e-b53a-9d838489d10a/en-14662-1-2005 accessed on 25 July 2023). 

### 2.4. Exposure Assessment and Environmental Burden of Disease (EBD)

The degree of air pollution has a direct and proportional impact on human health [21]. In this matter, an assessment of the exposure and environmental burden of disease (EBD) was performed for the conditions of Ploieşti City. We used EPA’s Exposure Factors Interactive Resource for Scenarios Tool (ExpoFIRST v. 2.0) (https://cfpub.epa.gov/ncea/risk/recordisplay.cfm?deid=322489 accessed on 25 July 2023) for computing the inhalation Average Daily Dose (mg/kg-day) (ADD) and Lifetime Average Daily Dose (LADD). 

Selections were air media type, inhalation rate type (long-term [daily]), and additional parameters related to location/activity and receptor characteristics (gender, age bins, and other population groups). Benzene was defined in the contaminants section by entering the contaminant concentration and units, permeability coefficient (0.14 cm/h under standard conditions at 26 °C [22]), and molecular weight (78.11184 g/mol). All the steps required for computation are described in [23]. The results section provides a summary of dose estimates by contaminant and receptor group based on the exposure scenarios that have been previously defined; then, the detailed results were exported to an MS Excel ExpoFIRST report (see Supplementary File S1).

Table S1 shows the main parameters used to define the scenarios for ADD and LADD. The corresponding equations are also described in Supplementary.

The health impact was estimated based on several indicators such as lifetime cancer risk (LCR), Hazard Quotient (HQ), Disability-Adjusted Life Years (DALY), and environmental burden of disease (EBD). We used the equations presented by Begou and Kassomenos [9] for the computation of the indicators, which were integrated in MS Excel (see Supplementary File S2). 

### 2.5. Statistical Analysis and Geospatial Modeling

Data inferences were performed using SPSS software (SPSS Inc., Chicago, IL, USA, 2011) and MATLAB (MATLAB and Statistics Toolbox Release 2012, The MathWorks, Inc., Natick, MA, USA). 

Descriptive statistics provided the main features of the dataset regarding the health and exposure variables determined in various sampling sites of the city. The t-test was used to evaluate the differences in means between years and between pre- and post-lockdown and lockdown in 2020. Correlations between the variables were tested using the Pearson product-moment correlation (*p* < 0.05). We used the IDW algorithm in ArcGIS Desktop 10.8.2 to obtain interpolation maps for benzene spatial distribution.

NOAA HYSPLIT dispersion model [24,25] was used for the assessment of dispersed concentrations, forward trajectories, and particle cross-sections based on GDAS1 meteorology and specific configuration of the model parameters (see Supplementary). In this work, a simulation of the maximum hourly concentration for benzene recorded on 29 July 2021 (4:00 a.m.) was selected for presentation. 

## 3. Results

### 3.1. Differences between Various Locations of the City and Its Suburbs

The analysis of the time series for each station showed the variation of the benzene concentrations in each location for the three years (Table 1). Overall, the annual average of all stations was almost similar between years i.e., 3.46 in 2019, 3.41 in 2020, and 3.63 µg/m^3^ in 2021, respectively. 

Regarding the spatial variation, the lowest multiannual average was in the east of the city 2.34 µg/m^3^ (PH-6 station), while the highest was reached in Brazi, at the PH-4 station that is located at 11 km in the south of Ploieşti (4.64 µg/m^3^). Interestingly, the other remaining stations had similar multiannual average values (3.4–3.6 µg/m^3^) suggesting similar conditions of dispersion due to local topography and meteorology (plain and wind regime).

The maximum hourly concentrations recorded between 2019 and 2021 were high, especially at Brazi station (PH-4), where the value was 190.29 µg/m^3^ in 2021. Most of the stations experienced a diminishing of the maximum values from 2019 to 2021, except the stations located outside the city. 

### 3.2. Differences between the Years Based on Aggregated Time Series (2019–2021)

**Figure 2:**
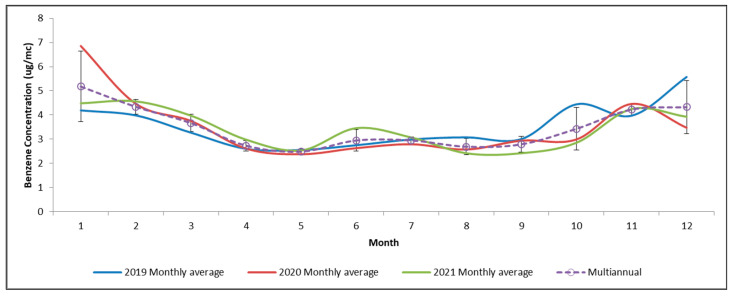
Benzene aggregated time series of monthly averages for all six stations (μg/m^3^) in the Ploieşti area between 2019 and 2021; error bars represent the standard deviation between years.

The time series of monthly averages for all six stations were aggregated to have a better image of the trends in the studied area. Figure 2 shows the dynamics of the benzene concentrations. 

Cold months tend to reach higher concentrations, compared to warm ones. In 2020, there was a slight reduction between April and July compared to the other two years potentially related to the lockdown restrictions. Based on the aggregated monthly averages, the lowest concentration of benzene was in May (~3 µg/m^3^), while the highest was in January (~5 µg/m^3^). The t-test results showed no significant difference in means between the pairs of years, i.e., 2019–2020 (t = 0.39; σ = 0.69), 2019–2021 (t = 1.34; σ = 0.18), and 2020–2021 (t = 0.89; σ = 0.37). Higher concentrations occurred in October and December 2019 and in January 2020, mainly because of longer periods of atmospheric calm that favored the stagnation of emissions. After January 2020, the time series from February to December 2020 was almost similar to the one from 2021.

**Figure 3:**
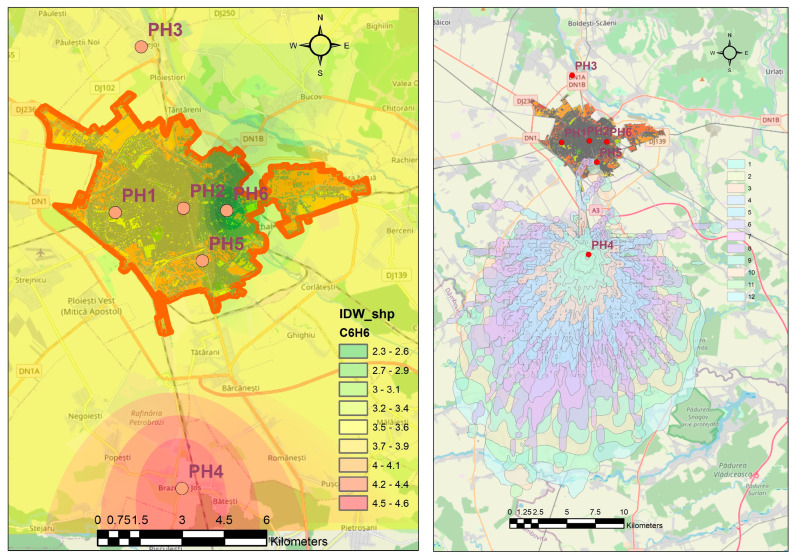
Simulation of the spatial distribution of benzene. Left: Inverse distance weighting algorithm results for the multiannual averages of benzene (µg/m^3^) based on the recordings from the six stations in Ploieşti City. Right: HYSPLIT dispersion results for the highest concentration of benzene (190.29 µg/m^3^) recorded on 29 July 2021 (4:00 a.m.) at the PH-4 station with layers computed every 25 min (1–12).

**Figure 4:**
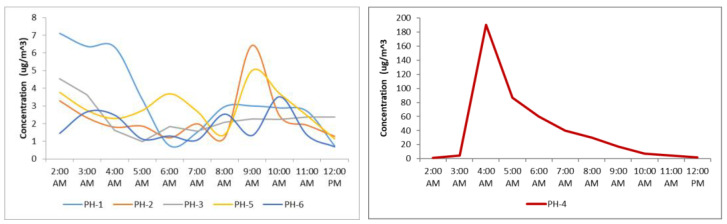
Evolution of the benzene concentration on 29 July 2021 between 2:00 and 12:00 a.m. during the maximum value of the period reached at the PH-4 station.

Figure 3 shows two case studies regarding the spatial distribution of benzene in the Ploieşti area. The first one presents the interpolation of the multiannual average concentrations showing that the southern area is more affected by the benzene presence. However, the entire area is experiencing similar concentrations. The second case study highlights the HYSPLIT dispersion results for the highest concentration of benzene (190.29 µg/m^3^) recorded on 29 July 2021 (4:00 a.m.) at the PH-4 station with layers computed every 25 min (3 h). It was noted that the dispersion of pollutants had an elliptic form oriented toward the south that did not affect the city. This was confirmed by the data recorded from the remaining stations, which showed usual values from 4:00 a.m. to 12 a.m. (at 12:00 a.m., the concentration at PH-4 reached 2.06 µg/m^3^) (Figure 4).

### 3.3. Differences in Concentrations during COVID-19 Lockdown in 2020

COVID-19 became a pandemic starting in March 2020, determining numerous restrictions on socio-economic activities, traveling, and commuting, and significant modifications in human behavior all over the world. In Romania, the lockdown period was considered between 13 March and 15 May 2020 based on the emitted official decisions [26].

**Figure 5:**
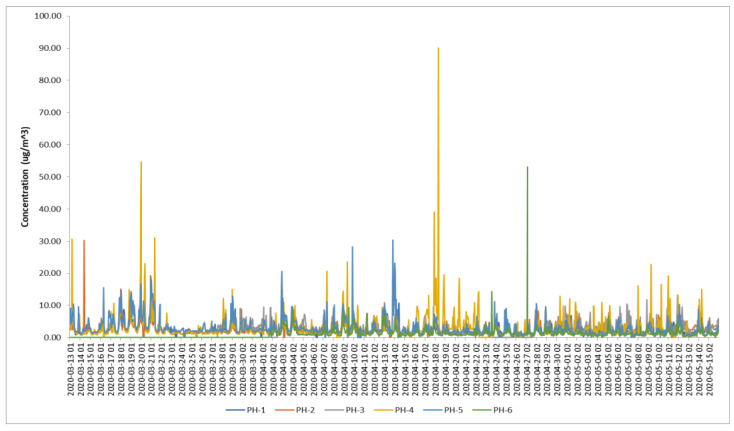

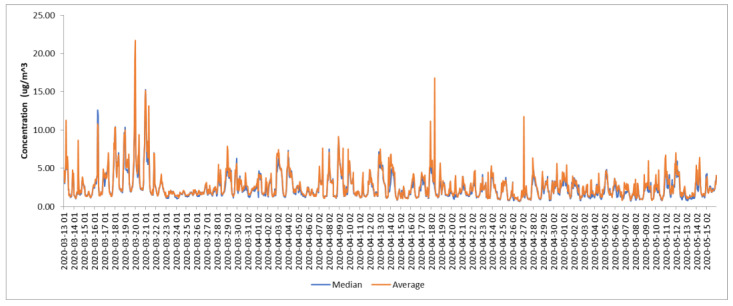
Benzene concentrations (μg/m^3^) during the COVID-19 lockdown established in Romania from 13 March 2020 when schools were closed, up to 15 May 2020 when the State of Alert replaced the State of Emergency [26] at all stations—hourly values with a daily scale tick mark. The second graph presents the aggregated median and average of all stations.

Figure 5 shows the time series of benzene concentrations recorded at the stations during the lockdown and the median and average of all of them. A drop in concentrations was observed starting from 23 March up to 28 March 2020. Then, other periods with reduced concentrations occurred between 5 April and 7 April, and between 13 May and 15 May. The dates coincide with Decrees and Military Ordinances that imposed more exigent restrictions on mobility (see [26] for detailed timelines and descriptions).

Table 2 presents the descriptive statistics of the time series recorded during the COVID-19 lockdown. The industrial stations (PH-4 and PH-6) showed the highest coefficients of variation and maximum values pointing out fluctuations related to the industrial activities, which were less affected by restrictions. The highest peaks were observed at the PH-4 station. On the other hand, the data captured at the PH-3 and PH-6 stations were around 69%, which might reduce the precision of the assessment. For a general assessment, the average of all stations during the lockdown period was 2.67 µg/m^3^, which was lower than the multiannual average of the 2019–2021 period (3.5 µg/m^3^) or compared to the average of individual years from Table 1. Another remark is that benzene was present in the air being recorded in each location where the analyzers of the stations operate, despite the restrictions on movement that reduced the emissions from traffic and filling stations. The lowest minimum value was recorded at the PH-3 station (suburban type) located outside the city i.e., 0.19 µg/m^3^.

A comparison between the differences in means of the lockdown and pre-lockdown (from 1 January to 12 March) and post-lockdown (from 16 May to 31 December 2020) using a *t*-test showed significant results for the pre-lockdown–lockdown (t = 32.06; σ = 0.00) and pre-lockdown–post-lockdown (t = 32.19; σ = 0.00) pairs. The difference in means of lockdown and post-lockdown was not significant (t = 1.84; σ = 0.06), but, interestingly, the average of the post-lockdown period in 2020 (2.55 µg/m^3^) was lower than the one recorded during lockdown.

### 3.4. Relationships with Other Pollutants and Meteorological Factors

**Figure 6:**
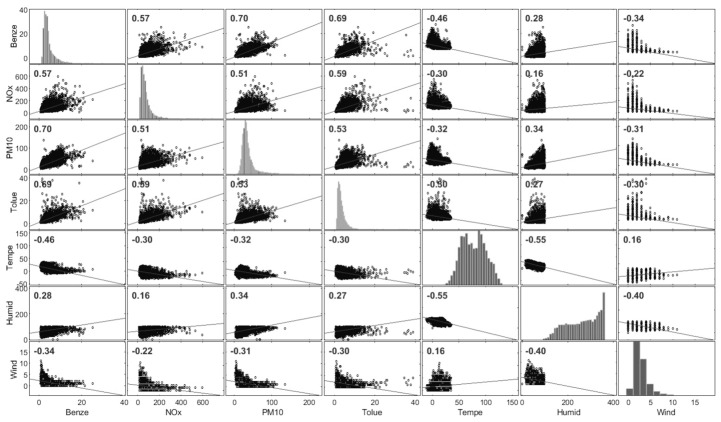
Correlation matrix of benzene with other pollutants and meteorological factors in Ploieşti City.

Figure 6 describes the correlations between benzene and other pollutants such as NOx (r = 0.57), particulate matter PM_10_ fraction (r = 0.70), toluene (r = 0.69), benzene and temperature (r = –0.46), humidity (r = 0.28), and wind speed (r = –0.34). Negative correlations (*p* > 0.01) were found between benzene and temperature and wind speed, respectively.

### 3.5. Benzene Patterns and the Potential Impact on Health in Ploieşti Area

Using the ExpoFIRST tool, the ADD and LADD (mg/kg-day) were calculated for three scenarios that may potentially occur. The first one considers a concentration of 3.5 µg/m^3^, which was the multiannual average value of the analyzed period. Eleven age bins were assessed by using the default parameters from the tool for an exposure frequency of 100 days/year for a better reference. The second scenario took into account a concentration of 6 µg/m^3^, which was recorded in Brazi at PH-4 in 2021, also considering default exposure and 100 days frequency. The third scenario was defined for a 3 h daily exposure in outdoor conditions (180 min) for all age bins (for example, for the category 21 years to <70 years, the default exposure is 283 min). The three hours of daily exposure was calculated as average daily current time spent outdoors (2 h × non-working day) + (3 ½ hours × working day)/7 for summer and winter and the mean of this was calculated to obtain a total daily current time spent outdoors [27]. More details related to the average time spent in outdoor conditions can be found in [27,28]. For the LADD, the exposure frequency was 365 days. Table 3 presents the results and Supplementary File S1 shows the output of the simulation for scenario 2. 

The simulations pointed out that the most affected age categories could be as follows:

In Scenario 1: 6 months to < 1 year (5.43 × 10^−4^ mg/kg-day), Birth to < 1 month (4.99 × 10^−4^), 3 years to < 6 years (3.87 × 10^−4^), and 21 years to < 70 years (3.58 × 10^−4^), respectively;

In Scenario 2: 6 months to < 1 year (9.31 × 10^−4^ mg/kg-day), 3 years to < 6 years (6.63 × 10^−4^), 21 years to < 70 years (6.13 × 10^−4^) and 6 years to < 11 years (5.69 × 10^−4^), respectively;

In Scenario 3: Birth to < 1 month (8.99 × 10^−4^ mg/kg-day), 1 year to < 2 years (8.41 × 10^−4^), 2 years to < 3 years (7.73 × 10^−4^) and 1 month to < 3 months (7.11 × 10^−4^). 

Overall, in all scenarios, the most affected age categories are small children, despite a lower outdoor exposure time.

Regarding the LADD, the highest value (10.6 × 10^−4^) occurs in scenario 2, then scenario 3 retrieves 6.76 × 10^−4^, and scenario 1 retrieves 3.95 × 10^−4^ mg/kg-day, respectively.

**Figure 7:**
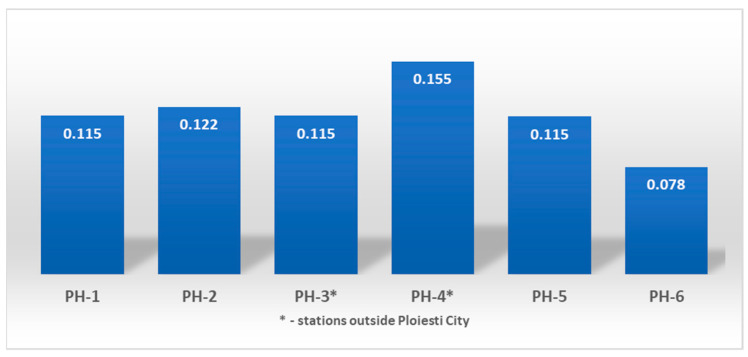
Hazard Quotient (HQ) computed for the analyzed period (2019–2021) for each station from the Ploieşti area.

Figure 7 shows that the HQ is lower than the limit value of 1 for all air quality monitoring stations from the Ploieşti area, indicating an acceptable level of exposure because the dose level is lower than the reference concentration (RfC = 0.03 mg/m^3^). The highest HQ for the exposed population was computed for PH-4 15.5 × 10^−2^ and the lowest was retrieved at PH-6 with a value of 7.8 × 10^−2^. Therefore, the residents’ health may not be affected by the potential non-carcinogenic effects of short-term exposure to ambient benzene.

The U.S. EPA estimated a range between 2.2 × 10^−6^ and 7.8 × 10^−6^ as the increase in the lifetime risk when an individual is continuously exposed to 1 µg/m^3^ of benzene in the air over his lifetime. Table 4 shows the calculated monthly and seasonal ILTCR (Integrated Lifetime Cancer Risk) values to assess the potential lifetime cancer risk. The seasonal variation of the period was characterized by values of 14.1 × 10^−5^ in winter, 9.04 × 10^−5^ in spring, 8.74 × 10^−5^ in summer, and 10.6 × 10^−4^ in autumn. The ILTCR annual averages were 1.08 × 10^−4^ (2019), 1.07 × 10^−4^ (2020), 1.04 × 10^−4^ (2021), and 1.06 × 10^−4^ for the entire period. 

For interpreting the results, the U.S. EPA has classified the risk intervals for the ILTCR as follows: >1 × 10^−4^ “definite cancer risk”; from 1 × 10^−5^ to 1 × 10^−4^ “probable cancer risk”; from 1 × 10^−5^ to 1 × 10^−6^ “possible cancer risk; and <1 × 10^−6^ “acceptable”. The resulting ILTCR values point out very risky conditions with the annual averages reaching the definite cancer risk category. 

In Romania, there is no exact situation regarding the incidence and mortality of hematological diseases and there are no official functional registers that could provide this information. The number of deaths in 2020 was 1277, most likely attributed to cases of acute leukemia (https://www.uicc.org/news/globocan-2020-new-global-cancer-data accessed on 27 July 2023). The 5-year prevalence is 27.37 per 100,000 inhabitants. We defined a scenario considering the number of 29 deaths from leukemia at a total population of 180,000 inhabitants, resulting in a DALY of 378.8 for a standard life expectancy of 72.8 years (EUROSTAT-https://ec.europa.eu/eurostat/statistics-explained/images/0/02/Table01_Life_expectancy_at_birth_2021.png accessed on 27 July 2023). Years lived with disability (YLD) was not included in the calculation of DALY by considering all cases of leukemia to be fatal, and consequently, the YLD equaled zero [9] (Supplementary File S2). 

Table 5 provides the results of four concentrations, 2, 3.5, 4, and 6 μg/m^3^ simulations for EBD, PAF, and DALY_EBD_. These figures were selected because they occurred during the analyzed period. The EBD estimates based on the number of excess deaths from benzene-related leukemia varied between 0.04 and 0.12, and the corresponding burden based on DALYs lost due to leukemia in Ploieşti was 0.291 (2 μg/m^3^ benzene), 0.509 (3.5 μg/m^3^ benzene), 0.582 (4 μg/m^3^ benzene), and 0.873 DALYs per 100,000 inhabitants (6 μg/m^3^ benzene), respectively. 

## 4. Discussion

The analysis of the air quality dataset showed that the benzene concentrations were significant in the Ploieşti area during the analyzed period (2019–2021). The multiannual aggregated average of the period was 3.5 µg/m^3^. Compared with the figures retrieved from the literature (Table S2), there are few cities that experienced higher values (e.g., Rome, Shanghai, Athens, Delhi) [9,18,29,30,31,32,33,34,35,36,37,38,39,40,41,42,43]. However, if the annual average of 6.14 µg/m^3^ recorded at Brazi, near Ploiesti in 2021, is considered, then the area is under a heavy impact on benzene and other VOCs. Even during the COVID-19 lockdown, the concentrations did not drop consistently for a longer period. The average of the period was approximately 2.7 µg/m^3^. 

Another issue was the occurrence of high concentrations of benzene for short periods. At a normal air temperature gradient (gradual cooling as the altitude increases), gases and dust have an upward evolution and are subject to an accentuated dilution. In the case of thermal inversions, the layers of colder air, blocked below the warm air, prevent the formation of convection currents (ascents) and block the emitted pollutants, which disperse horizontally, in situations of atmospheric calm, slowly draining towards the points lower where they form large accumulations. These inversions, which often occur in the area, especially during cold months, favor the phenomenon of atmospheric pollution and excessive exposure of the population [44,45,46,47]. 

The results showed negative correlations between benzene concentrations and temperature and wind speed, which explains a reduction of ambient concentrations when temperature and wind speed increase. Temperature has a marginally significant influence on the dispersion of benzene and other VOCs, which is stronger than the relative humidity effect [48]. During the daytime, Dayan et al. [49] found that elevated concentrations were found to be associated with the high prevalence of weak winds. In our research, a positive correlation is related to relative humidity but weaker compared to temperature and wind speed. Overall, the meteorological conditions have an important influence on benzene levels in urban areas.

The local newspapers often describe such pollution episodes signaled by the citizens from the impacted areas. According to the official data [50], 85% of the population of the Ploiești area believes that air pollution is the main cause of health problems and comfort dissatisfaction. It must be noted that data captured at some stations is not always sufficient, which means that the concentrations can reach high values that are not recorded and, thus, the “image” of the real status of the exposure is not sufficiently assessed. Since the stations from the national network are the only sources of information, by continuous monitoring, there is no other possibility to survey independently the VOCs in the area and provide public reports. Continuous monitoring of the emissions from the refineries existing in the area is important [51]. 

Based on the computation performed in this work, we found increased the ADDs and LADDs, especially for the children category, and a concerning incremental probability of an individual developing cancer over a lifetime exposure to the potential carcinogen being much higher than the U.S. EPA standard of 1 × 10^−6^ (i.e., 1 in 1,000,000 chances of developing cancer in the lifetime) [3].

In 2018, which is the latest public report, the mortality in Ploieşti from malign tumors was 142 per 100,000 inhabitants and 28 from respiratory malign tumors (15–64 years age bin), and 359 per 100,000 inhabitants and 78 (>65 years), respectively [52]. 

The Romanian Association against Leukemia (https://www.aril.ro accessed on 27 July 2023) is helping the patients and this help could be supported by proper legislative actions. A recent law (No. 293/2022) was enforced in Romania for the prevention and fight against cancer [53], which hopefully will improve the data hiatus in the following years. 

In March 2021, GLOBOCAN reported approximately 1900 new cases in Romania for leukemia, without distinguishing between acute and chronic forms, myeloid or lymphoid for the year 2020 [49]. 

Statistics from other countries report an average incidence of chronic lymphocytic leukemia (CLL) of 4.2 per 100,000 inhabitants/year (https://gco.iarc.fr/ accessed on 27 July 2023). Extrapolating the data to the country’s population, we can estimate a number of approximately 800–1000 new cases per year, corresponding to an incidence of 4.44–5.55 per 100,000 inhabitants/year [53].

Currently, in Romania there is no public information related to the specific mortality rate for patients, the rate of treated patients, the types of treatment, and their progression-free survival, data that are necessary to assess the management of patients in Romania. This extremely useful data could be obtained from a dedicated national disease registry. 

Recent studies underlined the key role of benzene in determining harmful effects on residents of refinery areas related to hematological and hypersensitivity fields, pathological spheres only seemingly disconnected from each other [54], and the existence of positive associations of cumulative levels of benzene, toluene, and xylene with the decline in monocyte counts, lymphocyte counts, and hematocrit of petrochemical workers [55]. More studies based on complete datasets are required to understand the magnitude of the impact of the elevated benzene concentrations, especially in the petrochemical areas. For example, in Delhi, India, the lifetime cancer risk values for males and females were higher during the lockdown period compared to the pre- and post-lockdown periods due to longer exposure time to increase the production of plastic and resin manufacturing units during the pandemic period [56].

The current study provides useful insights for a better understanding of the exposure levels to benzene and the associated health impact in Ploieşti, despite the limitations determined by the data hiatus and incomplete or missing information regarding the health impact. Future work will consider an assessment of the multipollutant effects on health by including other VOCs in the analysis [57].

## 5. Conclusions

The present study pointed out that the Ploieşti area was affected by elevated concentrations of benzene that impacted the health of the residents. Overall, the computations showed an increased risk of developing cancers and a relevant environmental burden of disease. Consequently, the air quality plan should be improved by following several guidelines:

- Initiation of a public register with data regarding the cases with diagnostics related to air pollution;
- Application of the best measures and technologies to reduce air pollution and protect the health of the population in areas with increased industrial pollution;
- Informing the population about air quality and individual protection measures;
- Reducing the level of pollution generated by road traffic and encouraging green technologies.

## Figures and Tables

**Table 1:** The annual variation of the benzene concentrations (µg/m^3^) in each of the six monitoring stations during 2019–2021 (*—stations outside Ploieşti City)–*N* = 365.

Indicator		Annual Average				Minimum			Maximum	
Year	2019	2020	2021	Multiannual	2019	2020	2021	2019	2020	2021
PH-1	3.34	3.63	3.42	3.46	0.32	0.09	0.41	33.57	41.54	31.06
PH-2	4.15	3.45	3.34	3.65	0.21	0.6	0.47	64.82	43.88	38.37
PH-3 *	4.11	3.37	2.91	3.46	0.03	0.19	0.01	34.1	36.8	37.88
PH-4 *	3.76	4.01	6.14	4.64	0.5	0.29	0.01	69.94	145.97	190.29
PH-5	3.46	3.32	3.54	3.44	0.48	0.59	0.31	103.15	42.57	37.91
PH-6	1.96	2.65	2.41	2.34	0.10	0.26	0.14	76.01	53.06	38.86
Average	3.46	3.41	3.63	3.50	0.31	0.35	0.24	63.60	60.64	62.40

**Table 2:** Benzene concentrations (µg/m^3^) recorded during the COVID-19 lockdown (13 March–15 May 2020) and main statistics at each of the six monitoring stations (*—stations outside Ploieşti City).

	N	Data Capture (%)	Average	Median	Minimum	Maximum	Std.Dev.	Coef.Var. (%)	Skewness	Kurtosis
PH-1	1509	98.6	2.4	1.96	0.3	12.45	1.52	63.13	2.02	5.72
PH-2	1510	98.6	2.62	1.98	0.8	30.18	1.96	74.82	4.49	38.98
PH-3 *	1056	69	3.34	2.91	0.19	13.32	1.99	59.63	1.19	1.67
PH-4 *	1473	96.2	3.04	1.93	0.49	90.03	4.23	139.35	9.27	147.2
PH-5	1512	98.8	3.14	2.31	0.64	30.31	2.68	85.41	3.57	20.19
PH-6	1055	68.9	1.48	1.02	0.27	53.06	2.01	135.71	16.84	415.04

**Table 3:** Average Daily Dose (ADD) (mg/kg-day) estimated for three scenarios based on 100 days exposure frequency for various age bins and Lifetime Average Daily Dose (LADD) (mg/kg-day) based on 356 days exposure frequency from birth to 70 years.

Indicator	ADD (mg/kg-Day)			LADD (mg/kg-Day)		
Simulation	Scenario 1 (3.5 µg/m^3^) Default Exposure Defined by ExpoFIRST)	Scenario 2 (6 µg/m^3^) Default Exposure Defined by ExpoFIRST	Scenario 3 (6 µg/m^3^) 3 h Outdoor Exposure, Daily	Scenario 1 (3.5 µg/m^3^) Default Exposure Defined by ExpoFIRST)	Scenario 2 (6 µg/m^3^) Default Exposure Defined by ExpoFIRST)	Scenario 3 (6 µg/m^3^) 3 h Outdoor Exposure Daily)
Age bin						
Birth to <1 month	4.99 × 10^−4^	4.28 × 10^−4^	8.99 × 10^−4^	-	-	-
1 month to <3 months	3.16 × 10^−5^	5.42 × 10^−5^	7.11 × 10^−4^			
3 months to <6 months	9.59 × 10^−5^	1.64 × 10^−4^	6.64 × 10^−4^			
6 months to <1 year	5.43 × 10^−4^	9.31 × 10^−4^	7.04 × 10^−4^			
1 year to <2 years	1.68 × 10^−4^	2.88 × 10^−4^	8.41 × 10^−4^			
2 years to <3 years	3.26 × 10^−4^	5.60 × 10^−4^	7.73 × 10^−4^			
3 years to <6 years	3.87 × 10^−4^	6.63 × 10^−4^	6.51 × 10^−4^			
6 years to <11 years	3.32 × 10^−4^	5.69 × 10^−4^	4.52 × 10^−4^			
11 years to <16 years	1.78 × 10^−4^	3.05 × 10^−4^	3.21 × 10^−4^			
16 years to <21 years	1.55 × 10^−4^	2.65 × 10^−4^	2.73 × 10^−4^			
21 years to <70 years	3.58 × 10^−4^	6.13 × 10^−4^	3.90 × 10^−4^			
Birth to <70 years	3.27 × 10^−4^	5.60 × 10^−4^	4.04 × 10^−4^	3.95 × 10^−4^	10.6 × 10^−4^	6.76 × 10^−4^

**Table 4:** Monthly and seasonal assessment of the lifetime cancer risk (ILTCR (Integrated Lifetime Cancer Risk)).

Year	2019	2020	2021	Season
January	1.28 × 10^−4^	2.09 × 10^−4^	1.37 × 10^−4^	
February	1.22 × 10^−4^	1.36 × 10^−4^	1.39 × 10^−4^	Winter
March	9.98 × 10^−5^	1.15 × 10^−4^	1.21 × 10^−4^	14.1 × 10^−5^
April	7.95 × 10^−5^	7.97 × 10^−5^	9.10 × 10^−5^	Spring
May	7.79 × 10^−5^	7.26 × 10^−5^	7.74 × 10^−5^	9.04 × 10^−5^
June	8.40 × 10^−5^	8.03 × 10^−5^	1.05 × 10^−4^	Summer
July	9.12 × 10^−5^	8.52 × 10^−5^	9.38 × 10^−5^	8.74 × 10^−5^
August	9.39 × 10^−5^	7.86 × 10^−5^	7.38 × 10^−5^	
September	9.17 × 10^−5^	8.97 × 10^−5^	7.39 × 10^−5^	Autumn
October	1.36 × 10^−4^	9.13 × 10^−5^	8.71 × 10^−5^	10.6 × 10^−4^
November	1.21 × 10^−4^	1.36 × 10^−4^	1.30 × 10^−4^	
December	1.70 × 10^−4^	1.06 × 10^−4^	1.20 × 10^−4^	
Average	1.08 × 10^−4^	1.07 × 10^−4^	1.04 × 10^−4^	1.06 × 10^−4^

**Table 5:** Environmental burden of disease (EBD) in the total population due to the exposure to benzene at various annual concentrations that occurred in the Ploieşti area (number of deaths from leukemia: 29; total population: 180,000; DALY: 378.8).

Annual Average Benzene Concentration (μg/m^3^)	EBD (Number of Excess Deaths)	PAF (Population Attributable Fraction)	DALY_EBD_	DALY_EBD_ per 100,000 Inhabitants
2	0.04	0.00138	0.524	0.291
3.5	0.07	0.00242	0.917	0.509
4	0.08	0.00276	1.048	0.582
6	0.12	0.00415	1.571	0.873

## Data Availability

The data presented in this study are available on request from the corresponding author.

## Supplementary Materials

The following supporting information can be downloaded at https://www.mdpi.com/article/10.3390/toxics11090748/s1, Supplementary File S1: ExpoFIRST modeling of the ADD and LADD (MS Excel File); Supplementary File S2: Computation of the HQ, ILTCR, DALY, EBD, etc. (MS Excel File); Table S1: Main parameters used to define the scenarios for ADD and LADD calculations in ExpoFIRST and corresponding equations; Table S2: Annual concentrations of benzene (µg/m^3^) retrieved from literature and results from the current study; Figure S1: Example of the LADD computation in ExpoFIRST (screen captures); Figure S2: Screen capture with an example of parameters calculation; Figure S3: Dispersion modeling for the maximum concentration of benzene recorded in Ploiesti area using the HYSPLIT model; Figure S4: Modeling of the particle positions using HYSPLIT Model; Figure S5: Forward trajectories provided by the HYSPLIT model.
