# Supplementary material for: A Three-Year Analysis of Toxic Benzene Levels and Associated Impact in Ploieşti City, Romania
Source: Toxics. 2023 Sep 2;11(9):748. doi: 10.3390/toxics11090748 (PMC10537639; doi:10.3390/toxics11090748)
Supplement: Supplementary file 1 [file toxics-11-00748-s001.zip › Supplementary.pdf]

Supplementary

A three-year analysis of toxic benzene levels and associated impact in Ploiesti City, Romania

Mia Sanda [1], Daniel Dunea [2,*], Stefania Iordache [3], Alin Pohoata [4], Ana-Maria Glod-Lendvai [2] and Ion Onutu [1]

Table S1. Main parameters used to define the scenarios for ADD and LADD calculations in ExpoFIRST and corresponding equations.

Variable	Selection/Input
Route of exposure	Inhalation
Exposure descriptor	central tendency
medium type	air
Location, activity category, or product category	Total outdoors
Gender	Male and Female
Manage contaminants	Molecular weight (78.11184 g/mol); Dermal permeability coefficient (0.14 cm/h)
Exposure Frequency (EF)	100 days (ADD); 365 days (LADD)
Exposure Time (ET)	Scenario 1 and 2 (default); Scenario 3 (180 minutes for all age bins)
ADD	*ADD = (C × InhR × ET × EF × ED) / (1440 (min/day) × AT × BW)*<br>where:<br>ADD - Average Daily Dose (mg/kg-day)<br>C - Concentration of contaminant in air (mg/m^3)<br>EF - Exposure Frequency (days/year)<br>ED - Exposure Duration (years)<br>AT - Averaging Time (days)<br>InhR - Inhalation Rate (m^3/day)<br>ET - Exposure Time (min/day)<br>BW - Body Weight (kg).
LADD	*LADD = (C × 1day × InhR × ET × EF × ED) / (24 hours × [BW] × AT)*<br>where:<br>LADD - Lifetime average daily dose (mg/kg-day)<br>C - Concentration of contaminant in air (mg/m3)<br>InhR - Inhalation rate (m^3/kg-day)<br>ET - Exposure time (hours/day)<br>EF - Exposure frequency (days/year)<br>ED - Exposure duration (years)<br>AT - Average time (days)<br>*For cancer effects, equals 70 years * 365 days/year*<br>[BW] - Body weight (kg) will be included in the denominator, if InhR is not normalized to body weight.

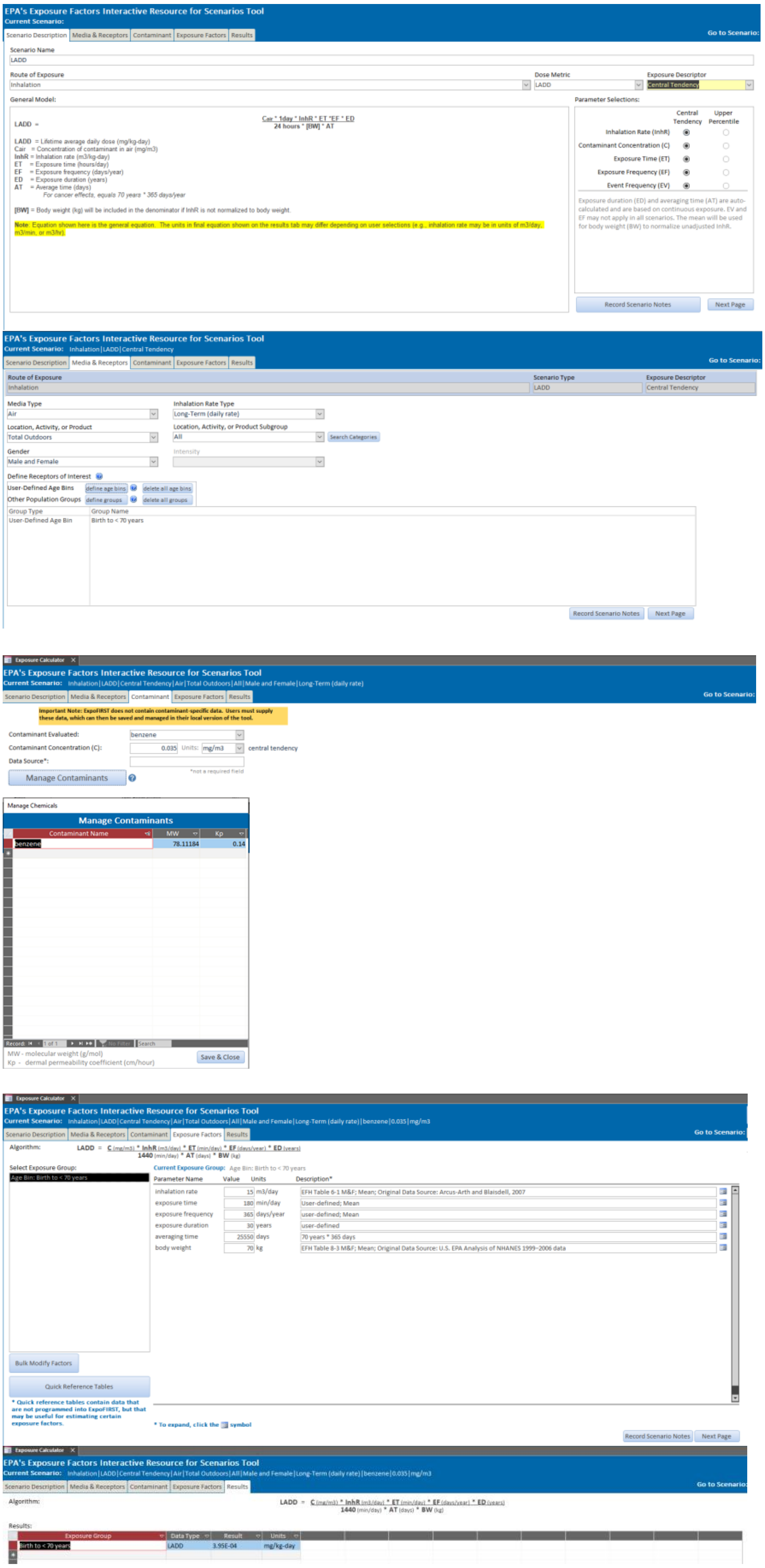

Figure S1. Example of the LADD computation in ExpoFIRST (screen captures)

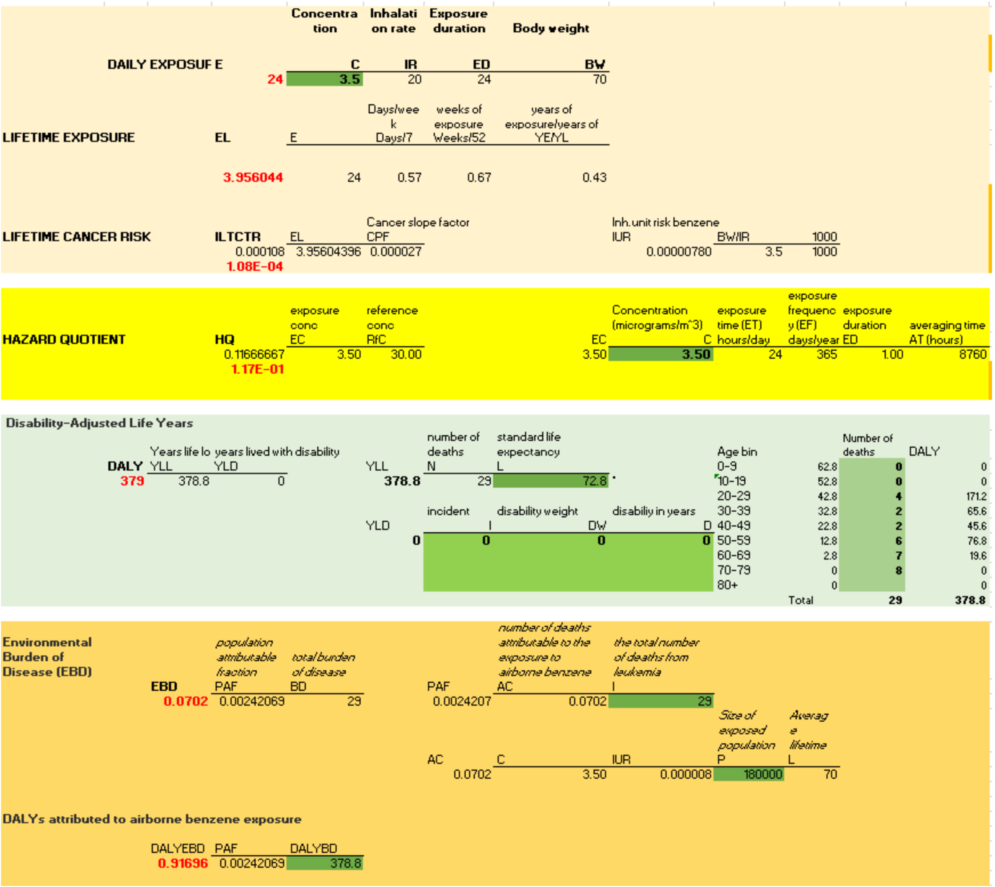

Figure S2. Screen capture with an example of parameters calculation (green cells are for inputs and red text highlights the values computed by the algorithms) – See Supplementary File 2 (excel file).

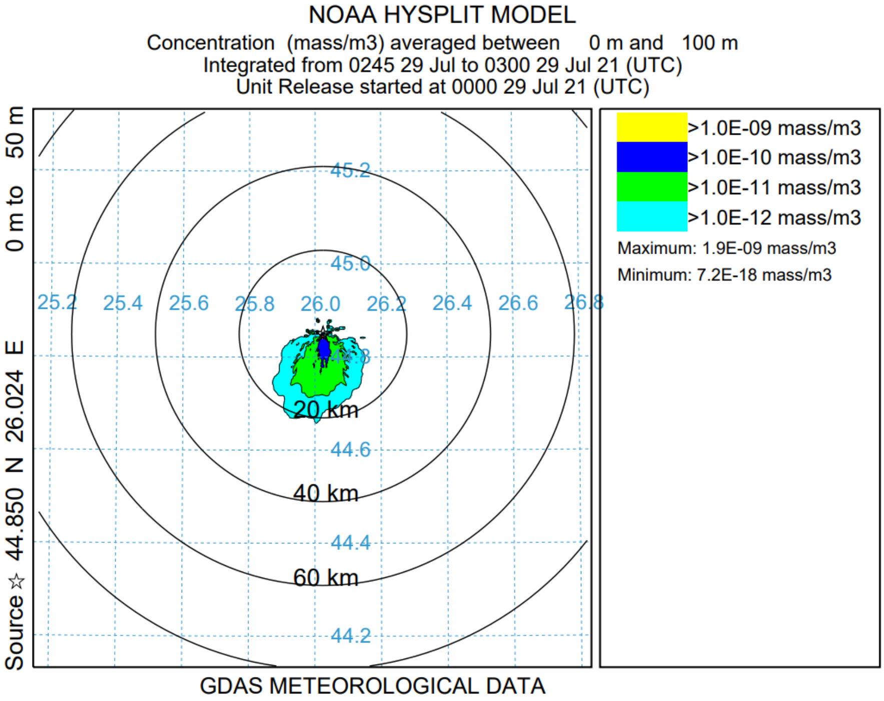

Figure S3. Dispersion modeling for the maximum concentration of benzene recorded in Ploiesti area using the HYSPLIT model

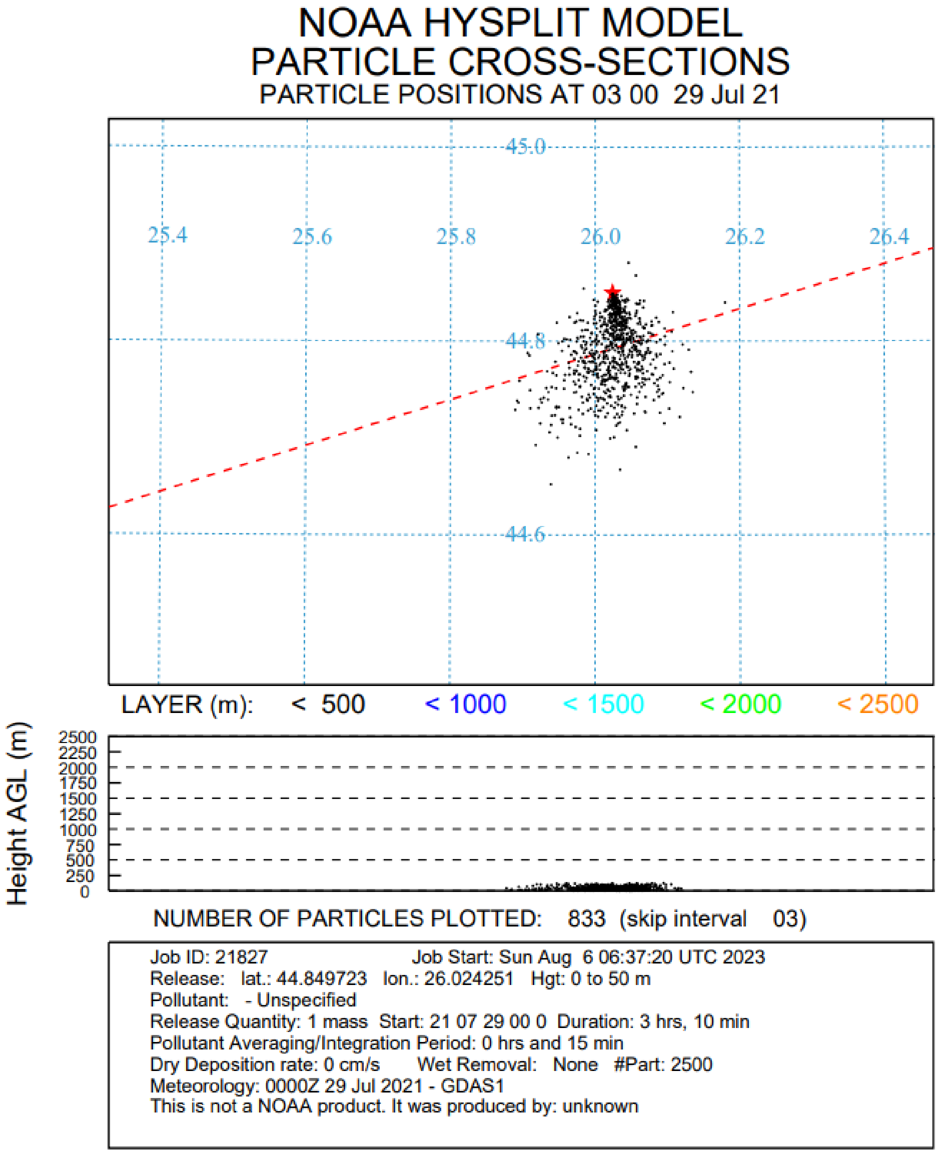

Figure S4. Modeling of the particle positions using HYSPLIT Model

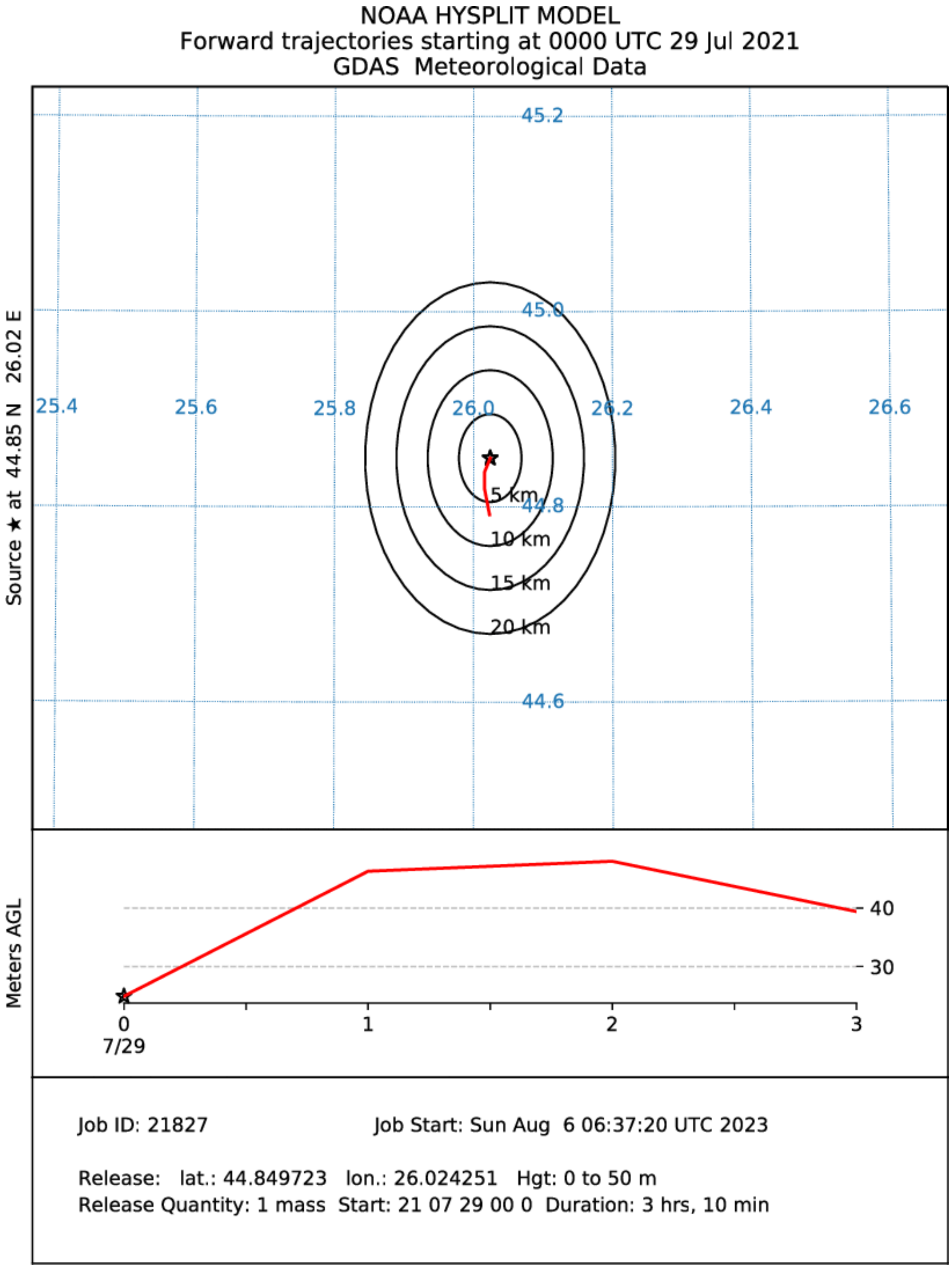

Figure S5. Forward trajectories provided by the HYSPLIT model

Table S2. Annual concentrations of benzene (μg/m^3) retrieved from literature and results from the current study.

City	Country	Type of Site	Benzene (μg /m^{-3})	Source
Berlin	Germany	Urban region	2.09	Monod et al, 2001 [29]
Bucharest	Romania	Urban region	1.75	
Krakow	Poland	Urban region	2.43	
London	U.K.	Urban region	0.8	
Prague	Czech Republic	Urban region	2.46	
Rome	Italy	Urban region	3.62	
Vienna	Austria	Urban region	0.99	
Warsaw	Poland	Urban region	0.75	
Paris	France	Urban region	1.42	
Hong Kong	Special Region of China	Urban region	1.64	
Lanzhou,	China	Urban region	1.94	Jia et al., 2016 [30]
Wuhan		Urban region	1.7	Lyu et al, 2016 [31]
Guangzhou		Urban region	2.4	Liu et al., 2008 [32]
Shanghai		Industrial (petrochemical area)	6.41	Zhang et al., 2018 [33]
Changzhi		Industrial	1.40	Zhang et al., 2022 [34]
Houston	USA	Urban region	0.34	Jobson et al., 2004 [35]
Naples Metropolitan area	Italy	Industrial	9	Iovino et al., 2009 [36]
Milan		Industrial	1.9 (Before lockdown)<br>0.96 (During lockdown)	Collivignarelli et al., 2020 [37]
Agii Theodore Corinthia	Greece	Industrial (oil refinery)	0.8	Kalabokas et al., 2001 [38]
Athens (Patission)		Urban (heavy traffic)	5.63	Begou and Kassomenos (2020) [9]
Piraeus		Harbor area	2.81	
Nea Smirni		Urban	1.68	
Elefsina		Urban-Industrial	0.81	
Thessaloniki (Agia Sofia)		Urban	3	
Kordelio		Urban-Industrial	2.14	
Sindos		Urban-Industrial	0.96	
Ulsan (industrial area)	Koreea	Industrial	2.1	Na et al., 2001 [39]

Port Moody	Canada	Industrial (petrochemical area)	0.6	Ying et al.,2020 [40]
Kaohsiung	Taiwan	Industrial (petrochemical area)	1.32-2.31 at Site A<br>1.65-3.30 at Site B	Hsu et al., 2022 [41]
Delhi	India	Industrial	6.08 (Before lockdown)<br>0.38 (During lockdown)	Pakkattil et al., 2021 [42]
Mumbai		Industrial	1.65 (Before lockdown)<br>0.47 (During lockdown)	
Bengaluru		Industrial	0.99 (Before lockdown)<br>0.332 (During lockdown)	
Shah Alam	Malaysia	Industrial	1.11+/- 0.870 (Before lock-down)<br>0.640+/- 0.350 (During lockdown)	Latif et al., 2019 [43]
Cheras		Industrial	1.05+/- 0.830 (Before lock-down)<br>0.640+/- 0.370 (During lockdown)	
Ploieşti	Romania	Urban, Industrial petrochemical area	3.25	Bodor et al., 2022 [18]
			3.5<br>2.7 (During lockdown)	(this study)
